# Supplementary material for: Association of Serum Albumin Levels and Long-Term Prognosis in Patients with Biopsy-Confirmed Nonalcoholic Fatty Liver Disease
Source: Nutrients. 2023 Apr 22;15(9):2014. doi: 10.3390/nu15092014 (PMC10180563; doi:10.3390/nu15092014)
Supplement: Supplementary file 1 [file nutrients-15-02014-s001.zip › nutrients-2309074-supplementary.pdf]

Association of serum albumin levels and long-term prognosis in patients with biopsy-confirmed nonalcoholic fatty liver disease

Hirokazu Takahashi, Miwa Kawanaka, Hideki Fujii, Michihiro Iwaki, Hideki Hayashi, Hidenori Toyoda, Satoshi Oeda, Hideyuki Hyogo, Asahiro Morishita, Kensuke Munekage, Kazuhito Kawata, Tsubasa Tsutsumi, Koji Sawada, Tatsuji Maeshiro, Hiroshi Tobita, Yuichi Yoshida, Masafumi Naito, Asuka Araki, Shingo Arakaki, Takumi Kawaguch, Hidenao Noritake, Masafumi Ono, Tsutomu Masaki, Satoshi Yasuda, Eiichi Tomita, Masato Yoneda, Akihiro Tokushig, Yoshihiro Kamada, Shinichiro Ueda, Shinichi Aishima, Yoshio Sumida, Atsushi Nakajima, Takeshi Okanoue, Japan Study Group of Nonalcoholic Fatty Liver Disease (JSG-NAFLD)
Supplementary Information

A. Supplementary Tables
Supplementary Table S1
Supplementary Table S2

Supplementary Table S1

Univariate unadjusted hazard ratios of death or OLT and liver-related death

	Death or OLT		Liver-related death	
**Variables**	HR (95% CI)	*P* Value	HR (95% CI)	*P* Value
**Albumin (g/dL)**				
High (>4.0)	1 (reference)		1 (reference)	
Intermediate (3.5–4.0)	3.58 (1.91–6.69)	**< 0.001**	11.2 (3.88–32.4)	**< 0.001**
Low (<3.5)	20.9 (7.72–56.8)	**< 0.001**	83.6 (21.1–330)	**< 0.001**
**Age >65 y**	2.58 (1.43-4.68)	**0.002**	2.45 (0.99–6.07)	0.053
**Male gender**	1.46 (0.75–2.81)	0.26	1.18 (0.49–2.85)	0.71
**BMI > 25 (kg/m^2)**	0.98 (0.52-1.87)	0.96	2.16 (0.63-7.40)	0.22
**NASH**	1.46 (0.75–2.81)	0.26	1.19 (0.46–3.10)	0.72
**DM**	1.75 (0.98–3.13)	0.057	2.40 (0.99-5.82)	0.053
**Stage**				
0	1 (reference)		1 (reference)	
1	0.73 (0.31–1.75)	0.48	0.88 (0.16–4.82)	0.88
2	0.92 (0.38–2.26)	0.86	1.84 (0.37–9.11)	0.46
3	2.29 (0.94–5.62)	0.07	5.22 (1.08–25.3)	**0.040**
4	1.19 (0.15–9.55)	0.87	4.81 (0.44–53.1)	0.199

CI, confidence interval; DM, diabetes mellitus; HR, hazard ratio: NASH, non-alcoholic steatohepatitis; OLT, orthotopic liver transplantation.

Supplementary Table S2

Multivariate adjusted hazard ratios for death or OLT

	Model 1		Model 2		Model 3	
**Variables**	HR (95% CI)	*P* Value	HR (95% CI)	*P* Value	HR (95% CI)	*P* Value
**Age >65 y**	2.49 (1.43–4.60)	**0.004**	2.69 (1.44–5.00)	**0.002**	2.42 (1.30–4.49)	**0.005**
**Male gender**	1.90 (1.05–3.44)	**0.034**	1.95 (1.07–3.55)	**0.029**	1.98 (1.09-3.60)	**0.025**
**BMI > 25 (kg/m^2)**					0.91 (0.47-1.76)	0.79
**NASH**			1.49 (0.57–3.87)	0.41		
**DM**					1.44 (0.79-2.62)	0.23
**Stage**						
0			1 (reference)			
1			0.51 (0.18–1.46)	0.21		
2			0.62 (0.18–2.06)	0.43		
3			1.29 (0.37–4.45)	0.69		
4			0.47 (0.05–4.55)	0.52		

Significant results are highlighted in bold.

CI, confidence interval; DM, diabetes mellitus; HR, hazard ratio; NASH, non-alcoholic steatohepatitis; OLT, orthotopic liver transplantation.
